# Comparison of computational algorithms for simulating an electrospray plume with a n-body approach

**DOI:** 10.1007/s44205-022-00015-w

**Published:** 2022-10-07

**Authors:** Sebastian K. Hampl, Marshall T. Waggoner, Ximo Gallud Cidoncha, Elaine M. Petro, Paulo C. Lozano

**Affiliations:** 1 Department of Mechanical Engineering, McGill University, 817 Sherbrooke St W, Montréal, H3A 0C3 Québec Canada; 2 Sibley School of Mechanical and Aerospace Engineering, Cornell University, 130 Upson Hall, Ithaca, 14850 NY USA; 3 Department of Aeronautics and Astronautics, Massachusetts Institute of Technology, 125 Massachusetts Ave, Cambridge, 02139 MA USA

**Keywords:** Electrospray Propulsion, N-body simulation, Barnes hut tree code, Fast multipole method

## Abstract

In order to better evaluate the trade-offs between different simulation options for an electrospray thruster plume, we have developed a multi-scale n-body code to compute the evolution of a single emitter electrospray plume in the pure ionic regime. The electrostatic force computations in the simulation are captured through the use of three different computational algorithms with various degrees of approximation. The results of the simulations for a simple test case are compared in terms of computational speed and accuracy. The test case utilizes a single operating point (323nA) for a stable meniscus solution for the ionic liquid EMI-BF4 firing in the positive pure ion mode. Complex species and probabilistic fragmentation processes are neglected. An overview is provided of the trade-off between accuracy and computational speed for the three algorithms in the context of simulating the electrostatic interactions between particles. For a large number of particles, the faster algorithms show a significant reduction in computational time while maintaining a high level of accuracy with a proper choice of tuning parameters.

## Introduction

Electrospraying is commonly used to eject mass from conductive liquids. The electrospraying technique has been used in the small satellite electric propulsion field due to its innate compactness, simplicity, and scalability [1]. The most commonly used sources in electrospray propulsion include ionic liquid and liquid metal ion sources, which are known to spray pure ions, thus maximizing the charge to mass ratio and achieving a very high efficiency and specific impulse (up to 4000 s [2]). During electrospray pure ion emission, a liquid propellant meniscus emerges at the top of a sharp needle or capillary, which is at balance between electric surface tension and hydraulic stresses. The resulting equilibrium shape adopts a conical form, which is generally different from the Taylor characteristic solution, as it does not involve a perfectly conical electrode geometry, and needs a finite hydrostatic pressure drop to sustain the emission of current. At the apex of the meniscus, the electric fields are high enough ($\sim 10^9$ V/m) to lower the solvation energy barrier of the ions and therefore eject them through a process of electrically-assisted thermionic evaporation [3].

The optics of the ion beams in electrospray ion sources are crucial to assess the performance and lifetime of electrospray thrusters [4], for instance the prediction of plume deflection angles for grid erosion, or neutral impingement to the walls [5]. It also contributes to the thrust, specific impulse, and beam divergence efficiency. Such factors are determined by the structure of the electric field across the ion acceleration region, the initial conditions of the ions right before emission, and the process of ion fragmentation. These electric fields typically span over multiple scales, from the $10^9$ V/m in the emission region to almost vanishing fields in the field-free region, and are a direct byproduct of the geometry of the acceleration region, including the features of the meniscus shape itself [6] and the curvature of the tip electrodes. The study of these processes affecting ion plume trajectories has motivated the development of multi-scale simulation frameworks at different levels of accuracy: from kinetic approaches [7] to particle-in-cell (PIC) models [8, 9]. The former model improves the accuracy of the electric field estimation by computing the exact Coulomb force between the particles at the expense of an intense computational scalability $\mathcal {O}(N^2)$^1^, where *N* is the number of particles being simulated. This $\mathcal {O}(N^2)$ scalability commonly limits the number of particles being simulated to $N \sim 10^4$, which corresponds to the number of ions that an ILIS source operating at $\sim 200$ nA ejects in 20 nanoseconds. The latter approach, PIC, uses a macro-particle approach and field grid interpolation, which can trade extended simulation times at the expense of a lower accuracy of the background electric field calculations as well as the inability to capture particle-particle interactions directly.

In this work, we implement algorithmic updates to the n-body method to alleviate the computational load and extend the simulation beyond the tens of nanosecond timescale. The algorithms include the Barnes and Hut octree division (BH) [5, 10] and the fast multipole method (FMM) [11]. The algorithms reduce the computational load from the direct N-body computation at $\mathcal {O}(N^2)$ to $\mathcal {O}(N\log {N})$ and $\mathcal {O}(N)$ respectively. As observed in previous iterations of the simulation framework, the inter-particle forces become a minor contributor beyond the close vicinity of the emission region ($\sim 5 \mu m$), and thus the direct method is computationally inefficient for many particle interactions. Hence, approximations are assumed to be justified without introducing any notable errors. The approximations are done by clustering groups of ions and approximating their far field contributions using multipole expansions. The details for each algorithm are provided at a later stage. This paper aims to assess the utility in the given simulation framework and analyze the trade off between accuracy and computational speed.

Previous works in the astrodynamic field have assessed the utility of publicly available, large galaxy, gravitational simulations with the same algorithms. The gravitational force computation is similar to the electrostatic force computation since both exhibit a $1/r^2$ force field and the force is calculated between all particles in the n-body simulation. For instance, [12] focuses on the computational aspects such as memory consumption and computational efficiency and the use of multi-core computing. Yokota et al. [13] developed and tested a generic BH and FMM code, as well as a hybrid Treecode-FMM and performed parameter tuning studies and error estimations as well as their performance on GPUs. Dehnen [14] focuses on assessing the accuracy of an advanced FMM code for gravitational simulations, its approximation error, and gives a guideline on how to minimize the computational effort at a given accuracy

In this paper, we take all of these aspects into account in the context of an electrospray plume, where interactions are governed by Coulombic forces and a background field. The work focuses on the practical aspects of chosing the correct algorithm for a given application and accuracy requirement.

This work is divided into five sections: After the introduction, in Section 2, the paper describes the underlying model for the single emitter plume and the test case that will be used to compare the algorithms. Section 3 is concerned with the algorithms and the type of approximations they make as well as the tuning parameters. In Section 4, the performance and accuracy are compared based on computational time, an error estimation, parameter variations, energy conservation, and specific particle trajectories. The last section, Section 5, provides a summary of the findings and points out gaps that can motivate future work.

## Model description and test case

### Overview and geometry

The simulation combines a fluid model and a particle model. The fluid model is adapted from [6] to handle curved geometries of the electrode. It contributes the background Laplace field, meniscus geometry and initial conditions of the particles at the beginning of their flight (position, velocity and current density, or probability of emission for a time step *dt*). The particle model integrates the particle trajectories across the background field and computes their mutual field interaction (Coulomb field) using a n-body approach. This calculation is performed with different algorithms, which are assessed for performance and accuracy in this work.

**Fig. 1:**
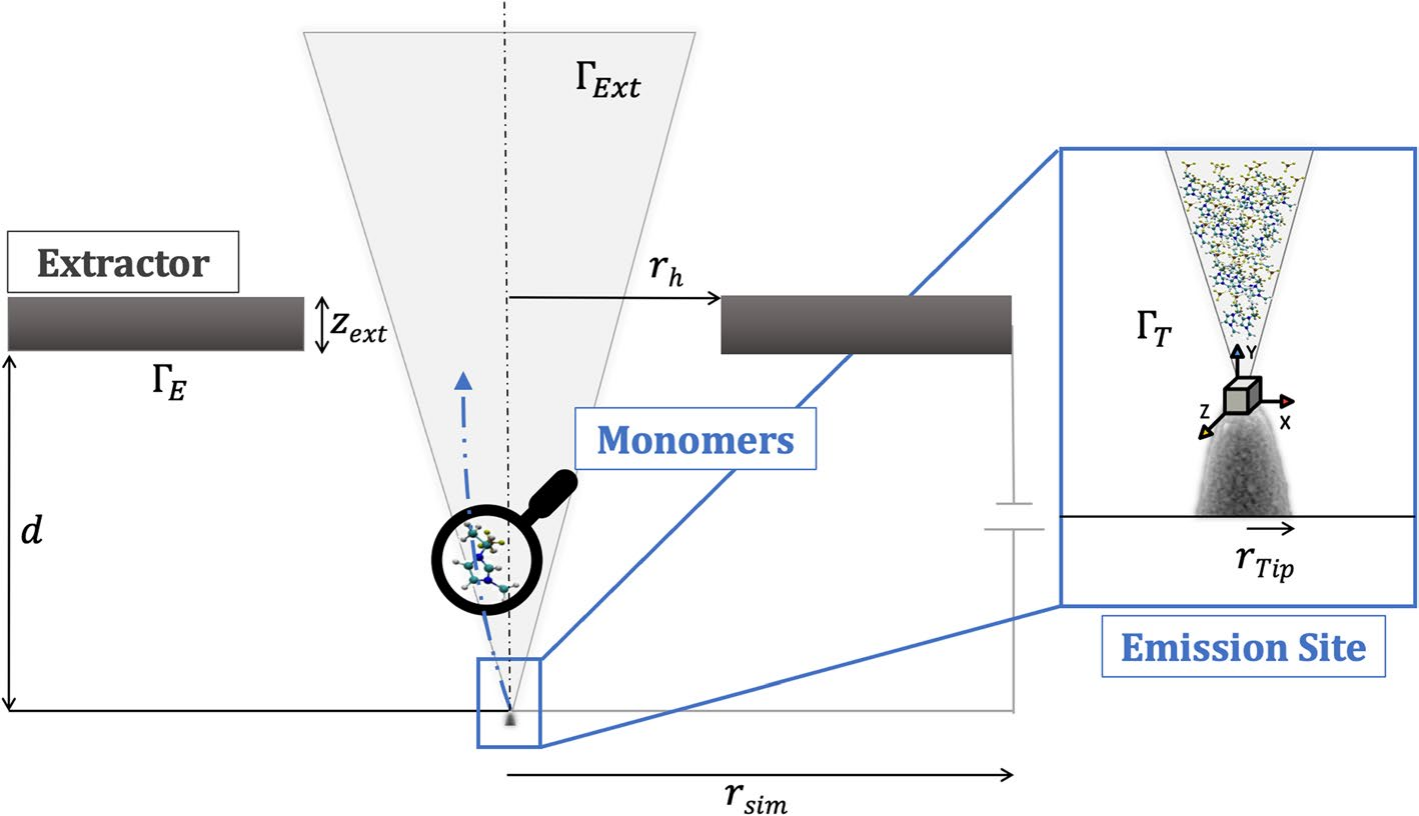
Computational domain of the simulation with its parameters. The values for the parameters are given in the text

Previous iterations of the n-body simulation framework [5, 7] include three types of ion species (monomers, dimers, and trimers) as well as a probabilistic fragmentation model. Moreover, the injection was randomized and hence probabilistic interactions and grid interactions were tracked. In order to produce consistent results and precisely compare the accuracy between the different algorithms, the code was simplified with the following assumptions:

- The injection procedure is pre-randomized for a subset of particles
- Only monomers are considered
- Fragmentation is neglected
- Particle interactions with the extractor grid are not tracked

The geometry however is the same as the one used in [7] and shown in Fig. 1. The ionic liquid flows through a porous needle (in practice oftentimes glass [15]) with flux $\Gamma _E$ and a potential of $\Delta \phi$ is applied. The flux to the extractor grid is $\Gamma _E$, which is modeled as a rectangular square with sides of length $l_{sim} = 300\mu m$ (the extractor covers entire downstream boundary except the extractor hole) and a thickness of $z_{ext} = 30\mu m$. The aperture in the middle of the extractor is $r_h = 150\mu m$ and the flux through it $\Gamma _{ext}$. The needle is modeled as a hyperboloid with linear eccentricity *a*, asymptote value $\eta _0$ and tip curvature $R_c = 11\mu m$. More details can be found in [7].

### Electrohydrodynamic modeling

The top of the tip contains an extrusion which acts a a fluid channel for the ionic liquid. The meniscus is in a stress equilibrium state and evaporates ions steadily along its surface. The equilibrium shape is computed from the EHD model based on the previously presented geometry and ionic liquid properties. Work by Coffman [16] and Gallud [6], present the model in detail. An adapted version of the Laplace equation is solved for the meniscus bulk and the particle acceleration region to handle ion charge flow through the interface. The equation includes a phenomenological law for current evaporation [3], which considers ion emission from the meniscus interface $\Gamma _M$ as an activated process regulated by the external electric field. In order to obtain the distribution of stresses on the mensicus surface, the Navier-Stokes and energy equations are solved. Then, when enforcing all constraints iteratively to achieve a balance of electric and fluid viscous stresses, the equilibrium shape can be calculated. When the shape is known, the other output parameters that serve as initial conditions for the particle model can be obtained. These parameters include the density map for the emitted current, initial velocity of the particles, and the Laplacian electric field solution for the emitting meniscus.

### N-body model and particle propagation

The n-body model integrates Newton’s second law to compute the particle trajectories through the domain after they have been injected from the meniscus surface. There is a contribution of the background Laplace field and the Poisson field, which is shown in Eq. 4. The calculation of the Poisson field and the algorithms will be discussed in the next section. The background electric field contribution is computed by interpolation from the mesh grids of the EHD model. A standard Delauny point search procedure is employed [17].

The integrator that is used in the simulation framework is a standard Leapfrog method which is second order accurate. The Kick-Drift-Kick (Eqs. 1, 2, 3) approach is used.

1 $$\begin{aligned} \mathbf {r}^{n+\frac{1}{2}}_i=\mathbf {r}^{n}_i+\mathbf {v}_i\frac{\Delta t}{2} \end{aligned}$$

2 $$\begin{aligned} \mathbf {v}_i^{n+\frac{1}{2}}=\mathbf {v}_i^{n}+\mathbf {a}_i\left( \mathbf {r}^{n+\frac{1}{2}}\right) \frac{\Delta t}{2} \end{aligned}$$

3 $$\begin{aligned} \mathbf {r}^{n+1}_i=\mathbf {r}^{n+\frac{1}{2}}_i+\mathbf {v}_i\frac{\Delta t}{2} \end{aligned}$$

### Implementation and codes

The model is implemented in a single code which allows the use of different initial conditions and fields as well as the specification of different integrators and algorithms which are described in Section 3. The Direct Force (DF) and BH algorithm as well as the electric field model and injection mechanism are adapted from previous work. The FMM algorithm was adapted from the open source GitHub repository of the Barba Group^2^, which is presented in detail in [11]. Other parts of the algorithms are adapted from Section 8.1 of [18]. Since those codes were primarily developed for gravitational simulations, both codes have been modified for simulating the electrospray plume and integrated with the electric field model and injection; proposed optimizations to the code including multi-threading or GPU computing were excluded for this study for reasons described earlier. Basic multi-threading capability (using pthread.h in C++) is written into the code, and the simulation is executed with a constant number of four threads. The effects of multi-threading and using GPUs for every type of algorithm proposed in this paper have been previously explored [12, 19].

### Test case

A total current of $I = 323$ nA is emitted with a maximum time step of $dt = 5$ ps which is well in the region of convergence for the time step as shown in [7]; all time steps below $1 \times 10^{-11}$ s are found to resolve the trajectory to within 0.1% accuracy. The applied potential is $\Delta \phi = 1823$ V and EMI-BF4 is used as the ionic liquid.

**Fig. 2:**
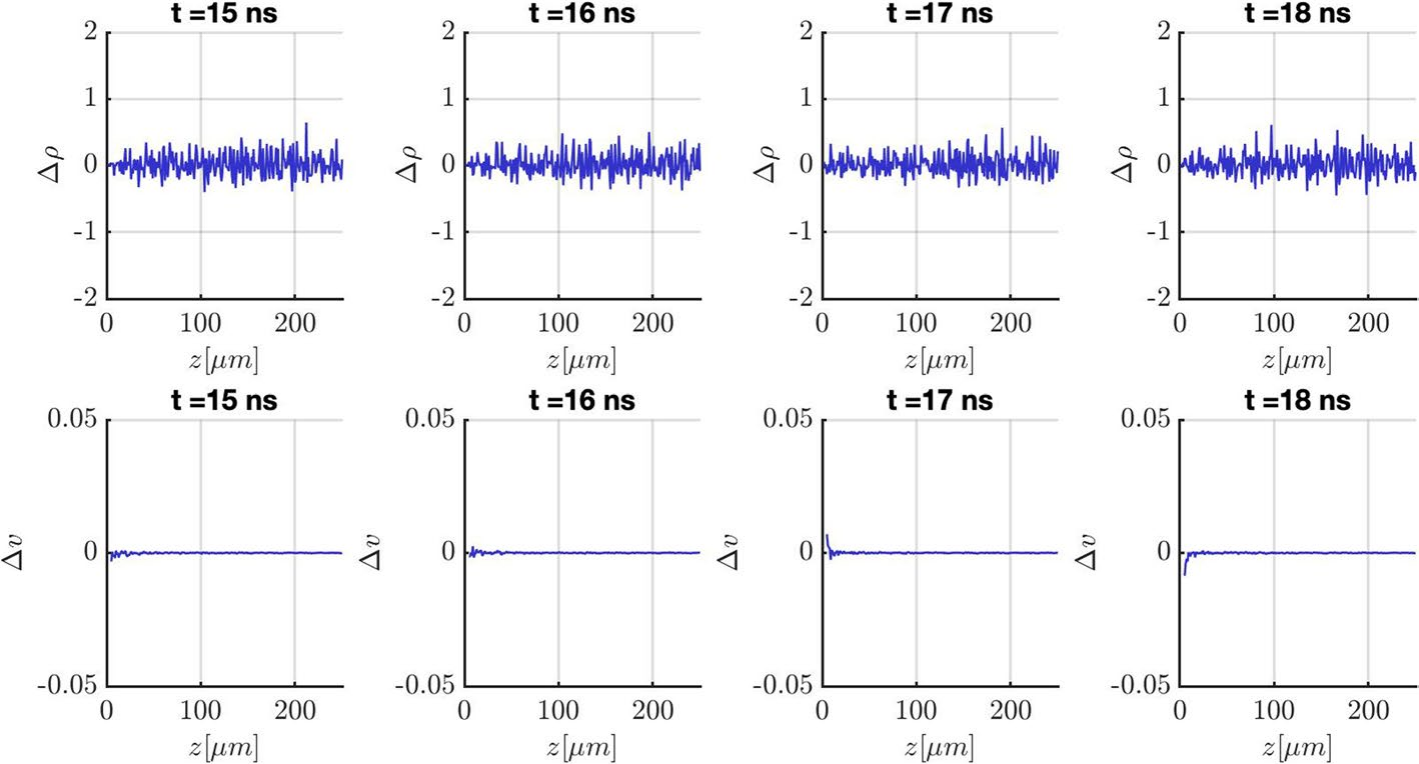
The change in velocity and density between discretized cylindrical plume segments along the z-axis of the simulation. The data is compared for snapshots at 15 ns, 16 ns, 17 ns, and 18 ns of a DF simulation

The steady-state requirement and the convergence in the time step is only relevant for the computation of the accuracy. Figure 2 shows the changes in velocity and density between discretized cylindrical sections of the plume along the z-axis for different snapshots in time. It can be observed that neither the density nor the velocity show significant changes for a simulation time of $\Delta t > 15$ ns between the cylindrical sections and therefore, the simulation can be assumed to be at steady-state in the whole domain.

**Fig. 3:**
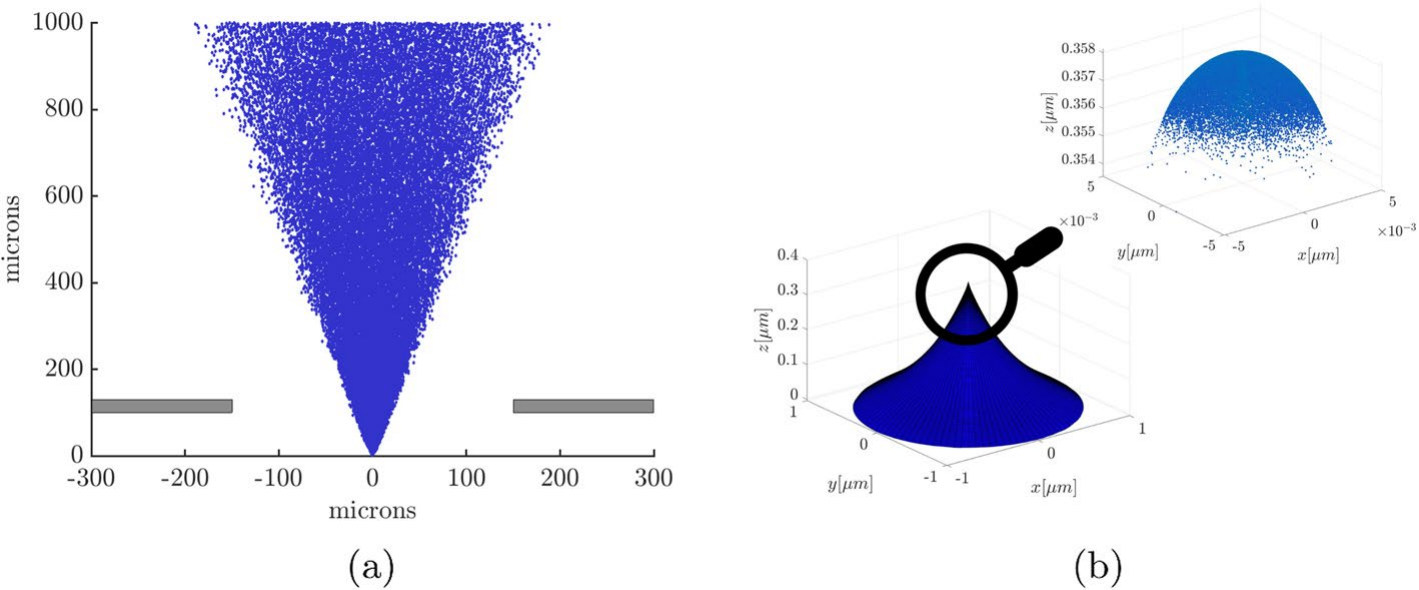
**a** Final state of the plume of a 343 nA direct force simulation with a simulation time of 20 ns and a maximum vertical propagation distance between a particle and the extractor of 1067 microns. **b** Initial distribution of the particles on the meniscus due to a higher emission probability on the top. The full shape of the meniscus is shown as a reference. The shape was obtained from the EHD model described in [7]

All simulations for which accuracy is compared are therefore run for at least 15 ns or longer. The beam will have propagated to approximately 1000 microns in the z-direction and particles are not deleted from the domain. The final state for an exemplary simulation is shown in Fig. 3a as well as the meniscus shape and the initial location of the particles on the meniscus before injection in Fig. 3b. The injection is randomized and pre-initialized for all particles that will be injected to ensure consistency within the different runs of the simulation. A current weighting is utilized to assign different probabilities of emission to different regions of the meniscus. As can be seen in Fig. 3b, there is practically no emission from the sides of the meniscus and the majority of particles is concentrated on the very tip of the meniscus.

## Algorithms

A short description of the governing equations of the different algorithms if provided below. Starting from the DF algorithm with the order $\mathcal {O}(N^2)$, the complexity is reduced to $\mathcal {O}(NlogN)$ in the BH Tree Code and then further reduced to the ideal $\mathcal {O}(N)$ using the FMM. As described in Section 2, the forces acting on a particle consist of a Laplacian background electric field $E_L$ and the particle-particle electrostatic interactions $E_C$. The equation is based on Newton’s second law for a charged particle of mass $m_i$:

4 $$\begin{aligned} m_i \mathbf {a}_i = q_i(\mathbf {E}_{L}(\mathbf {r}_{i})+\mathbf {E}_{C}(\mathbf {r}_{i},\mathbf {r}_{j})) \end{aligned}$$

The algorithms optimize the computation of the sum, which constitutes the second part of the equation. The Laplace field is not included in the explanation of the algorithms.

### Direct force method

**Fig. 4:**
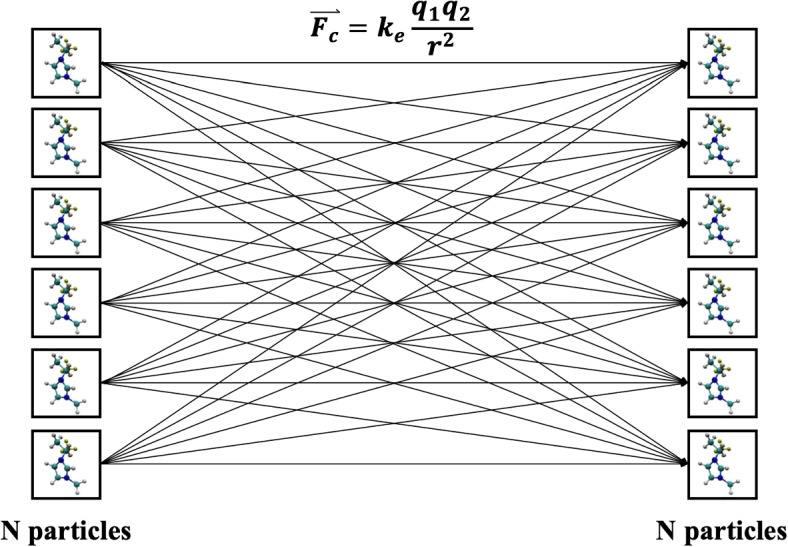
Illustration of the DF method

The direct force method method computes the computes the Coulomb force $\mathbf {F}_i$ directly as:

5 $$\begin{aligned} \mathbf {F}_i = \sum \limits _{j} \frac{q_i q_j (\mathbf {r}_{i} - \mathbf {r}_{j})}{4\pi \epsilon _0|\mathbf {r}_{i} - \mathbf {r}_{j}|^{3}} \end{aligned}$$

where $q_i$ is the charge of a particle interacting with particles of charges, $q_j$ and $||r_i - r_j||$ is the distance between the particles. An illustration is provided in Fig. 4. This method is of order $\mathcal {O}(N^2)$ and was implemented as a first iteration and tested by Petro et al. [7].

### Barnes-hut tree code

An approach to reducing the complexity of the calculation can be achieved by approximating the contribution to the force on the particle from distant particle clusters as the one created by a single large particle at the cluster’s center of charge. This approach is accurate as long as the cluster of particles is sufficiently far away from the particle for which the field is calculated.

The clustering is done recursively in a tree structure, such as the binary tree or the Barnes-Hut tree [10]. The efficiency of these tree structures has been studied from a computational perspective in previous works [19] with the BH tree being the most efficient one. This method is the one that Gallud, Petro et al. used in the second iteration of their code [5].

In the BH algorithm, the computational domain is discretized in octants (an hexahedral region of the three-dimensional space). The discretization is done hierarchically, where each of the N bodies are allocated in one of these octants (or nodes). The top of the tree is the root node that contains all the particles in the domain, while the children in the tree represent octants and contain the particles in the respective octant. Each parent node is recursively subdivided into children with a minimum of one particle required in each octant. In the simulation, the total charge and the spatial center of charge are computed and stored as part of the information about the child node. The Coulomb field is computed by traversing the nodes of the tree, starting from the root node. If the ratio $\theta$ between the size of the octant *s* and the distance between *d* between its center of charge and the particle ($\theta = s/d$) is sufficiently small (typically $\theta < 1$), then the approximated octree contribution to the force can be calculated:

6 $$\begin{aligned} \mathbf {F}_i = \sum \limits _{j} \frac{q_{i} q_{j} (\mathbf {r}_{i} - \mathbf {r}_{j})}{4\pi \epsilon _0|\mathbf {r}_{i} - \mathbf {r}_{j}|^3} \approx \frac{(\mathbf {r}_{i} - \mathbf {r}_{CM})}{4\pi \epsilon _0|\mathbf {r}_{i} - \mathbf {r}_{CM}|^3} q_i \sum \limits _{j} q_j \end{aligned}$$

In the equation, instead of $r_j$ for each particle inside the sum, the center of charge $r_{CM}$ for the octant is used. This approach reduces the complexity in an ideal case to $\mathcal {O}(NlogN)$, instead of computing each of the individual’s particle contributions.

**Fig. 5:**
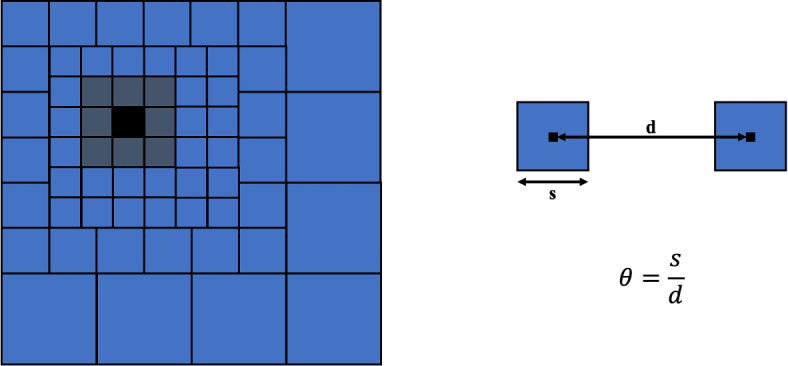
Schematic of how the force is calculated in the tree code for a given acceptance angle $\theta$. Each square represents an octant which contains at the minimum one particle

A schematic for the interaction criterion $\theta = \frac{1}{2}$ is shown in Fig. 5.

### Fast multipole method

The FMM adds another level of approximation to the code: instead of only clustering the particles in the far field, the FMM also considers cluster-to-cluster interactions and thus further reduces the complexity [20] which might enable us to simulate multiple emitters with significantly more particles in the domain. The approach of creating clusters is similar to the tree code but the force calculation is performed differently. Simply written, we partition the domain into cells, called P. Assuming that $i = 1,...,N$, where *N* is the number of particles and particle $i \in P_i$, we can create two sets:

7 $$\begin{aligned} S_1= & {} \{P:\;P\;is\;near\;to\;P_{i}\} \nonumber \\ S_2= & {} \{P:\;P\;is\;far\;from\;P_{i}\} \end{aligned}$$

**Fig. 6:**
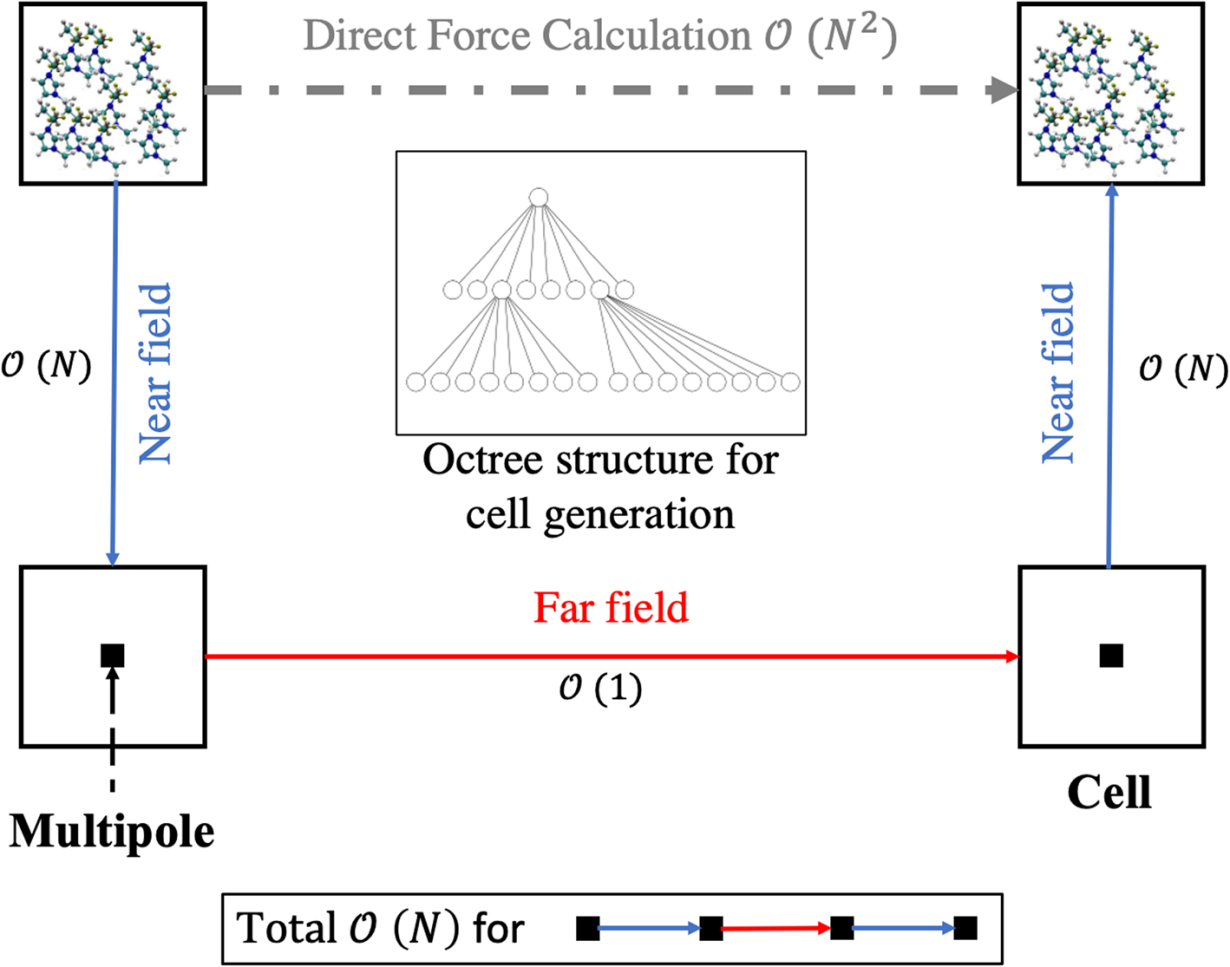
Simplified schematic of the FMM. For a more detailed graphic representation refer to [11]

Then, the potential can be approximated as:

8 $$\begin{aligned} \phi _i = \sum \limits _{P \in S_1}\sum \limits _{j \in P} M(\mathbf {r}_{i},\mathbf {r}_{j}) + \sum \limits _{P \in S_2}\sum \limits _{j \in P} M(\mathbf {r}_{i},\mathbf {r}_{j}) \end{aligned}$$

where *M* is a multipole expansion. The first set represents the near field interactions of the particles, while the second set is used to compute far field interactions. This distinction is schematically shown in Fig. 6 with the respective computational order of complexity. The example shows the four simple steps of the FMM force calculations for randomly located cells and neglecting the octree structure in the schematic. In general, the structure is the same as the BH tree, only the near field force computation is added as an additional step. The exact implementation and equations of the current code are adapted from Yokota and can be obtained in [11].

The seven key steps as well as the pre-processing procedure are extensively described and referenced in the Appendix as the concrete implementation of the method is not imminently relevant for the scope of this work.

Exact mathematical details, justifications for the approximations, and further details of implementation of Fast Multipole Methods can be found in [20–22].

### Computational time complexity

**Fig. 7:**
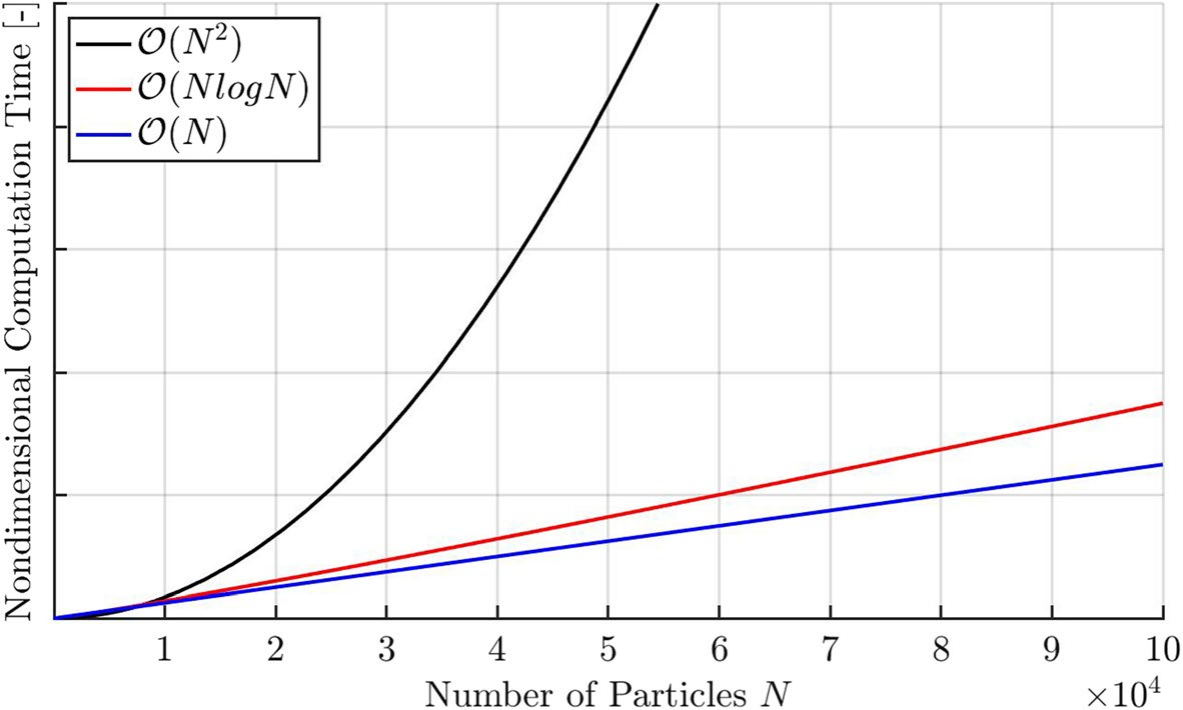
Schematic displaying the scaling of computation time with different orders of complexity in time

The computational time complexity scaling of the three algorithms has been mentioned previously. Figure 7 shows the scaling of the computational time with the number of particles *N* for $N < 10,000$ which is larger than the maximum number of particles simulated in this work. The y-axis is nondimensionalized as computation time varies on different computers while the trends stay the same. The advantage of using the faster algorithms especially for high number of particles is imminently visible.

### Key parameters

The key parameters that determine the accuracy of the two fast algorithms are the Multipole Acceptance Criteria (MAC), also called the acceptance angle, $\theta$, for the BH code and the order of expansion, *p*, for the FMM. In a tree code, the order of expansion *p* is kept constant ($p = 1$).

The MAC is defined as the ratio of the size of a cell *s* and the distance from the cell it is interacting with *d*, which is shown in Fig. 5. This criteria determines if the cell will be used to calculate the interactions or not; if the cell is small (small *s*), it will interact with cells at a closer distance, while a cell with a large *s* would only be accepted if it is further away. Changing the MAC alters the interaction list and thus the accuracy of the computations. A common range for $\theta$ lies between: $0 \le \theta \le 1$, where a value of $\theta = 0$ would be equivalent to a DF calculation. For a given value of *p*, a $p^{th}$-order multipole expansion is formed for each box about its center [23]. A higher choice of parameter *p* increases the accuracy, but also increases the computational time necessary. Typical values for the order of expansion are $1 \le p \le 20$ and the scaling of the computational time with the choice of parameter *p* is shown in [14].

Since the FMM method is often used in combination with a tree structure, a possible optimization of varying both parameters in a hybrid tree code-FMM as well as its implementation on a GPU has been explored [13]. To assess the accuracy of the codes separately and not introduce further complexity to the analysis, this work will not explore the joint variation of the two parameters.

## Performance and accuracy comparison

In this section, we compare the computational time, accuracy, and particle trajectories for the BH and FMM methods against the DF method for different values of the tuning parameters. We also analyze the impact of the different methods on the energy of the simulation. The simulations were performed on an Intel(R) Core(TM) i7-6500U CPU @ 2.50 GHz with 16.0 GB (15.9 GB usable) of installed RAM.

### Computational time

In order to compare the computational performance of the three different algorithms and the influence of the tuning parameters, different studies were performed. First, the influence of the tuning parameters for the BH tree code and FMM is investigated and afterwards the FMM and BH are compared. In every cases the DF method is used as a baseline. Computational errors, where singular simulations require a very long computation time due to for instance background processes are smoothed out through a moving median calculation. The background electric field calculation is part of the computational time but negligible (on the same order as the integration time; interpolation points are initialized before running the code, therefore the actual linear interpolation time is small) compared to the Coulomb force calculation time.

Particles are injected at each time-step, depending on the total current emitted *I*. The simulation time $\Delta t$ is directly correlated to the number of injected particles by

9 $$\begin{aligned} \Delta t = \frac{Nq}{I} \end{aligned}$$

where *N* is the number of particles, *q* the elementary charge, and *I* the current. The simulation time is plotted on all plots as a second x-axis.

**Fig. 8:**
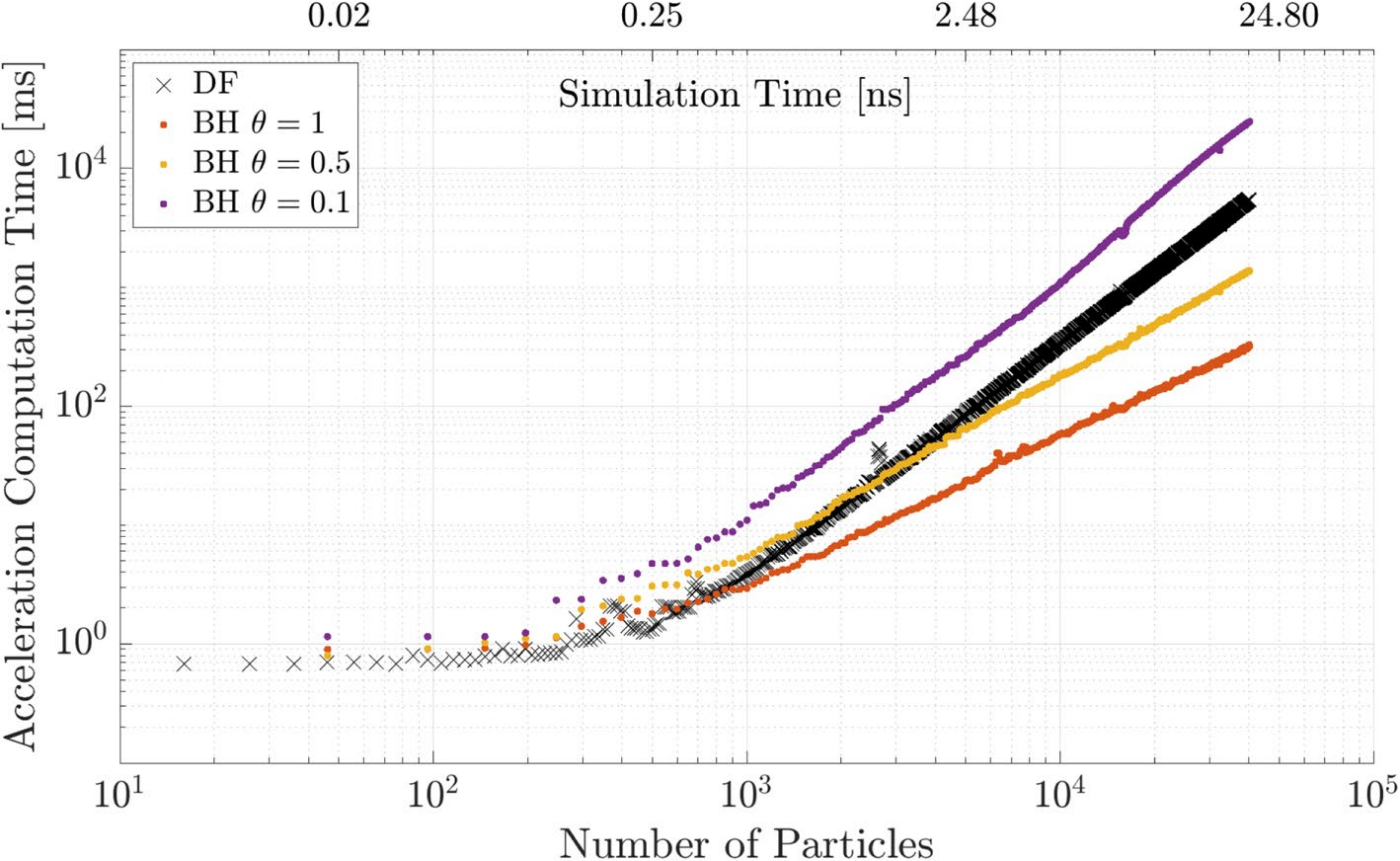
BH computation time for different MAC $\theta$ and comparison to the DF method

In a first simulation the BH tree code is run for three values of the MAC $\theta = \{0.1, 0.5, 1\}$ and the results are shown in Fig. 8. The computation time decreases for larger values of $\theta$ as expected. An important point to mention is that the BH method becomes faster than the DF method for $\theta = 0.5$ at around $N = 2000$ particles, while for $\theta = 0.1$ the DF method is always faster and for $\theta = 1$, the BH tree code becomes faster at approximately $N = 800$. The reason for the DF method being faster than a method with approximations is that the creation of the tree structures also requires computation time and the acceleration in the actual calculation only becomes visible for a larger number of particles.

**Fig. 9:**
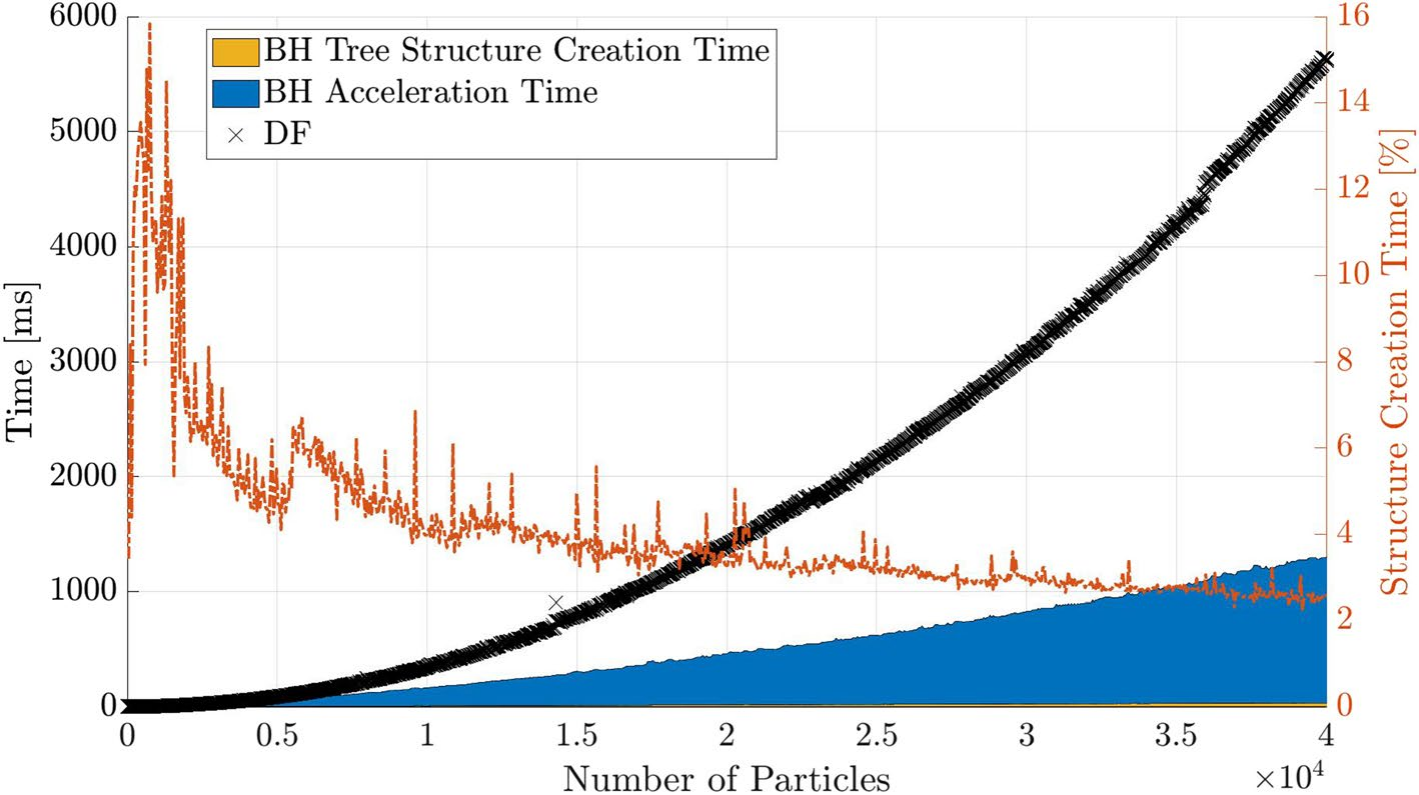
Computational time contributions for the BH method with a MAC of $\theta = 0.5$, separated into acceleration time and tree structure creation time. The right y-axis shows the contribution of the time needed to create the structure compared to the total time

The breakdown of tree structure creation and computational time for the BH method with $\theta = 0.5$ is shown in Fig. 9. The figure shows that the contribution of the tree structure creation time diminishes with a higher number of particles, and the force calculation time becomes the main factor which is significantly accelerated by using the approximation algorithm.

**Fig. 10:**
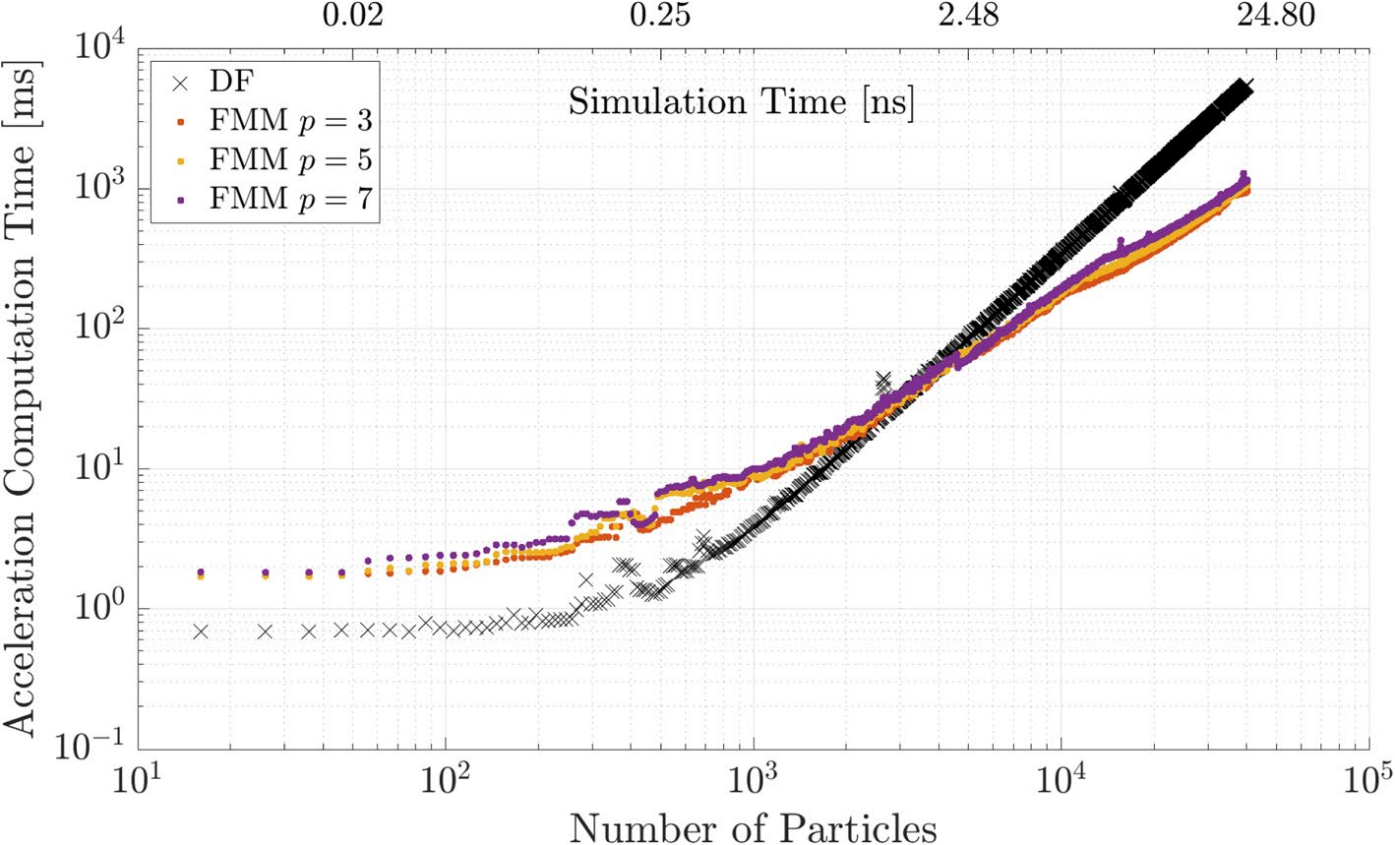
FMM computation time for different number of multipole expanions *p* and a comparison to the DF method

The same procedure is repeated for the FMM code and the multipole expansion criterion $p = \{3, 5, 7\}$. The results are shown in Fig. 10. Here, the DF method is faster for $N = 3000$ particles and after that the FMM becomes significantly faster, independent of parameter *p*. The computation time decreases with a lower *p*, which is expected since it means a higher degree of approximations. The difference when varying the parameters is less distinctive than for the BH method.

In the same manner as for the BH code, the computation time for the actual force computation and other parts of the algorithm are plotted for the FMM in Fig. 10. Since most of the algorithmic steps between particle-to-multipole (P2M) and multipole-to-particle (M2P) have negligible contribution to the computational time, they are combined into one part of the area^3^.

**Fig. 11:**
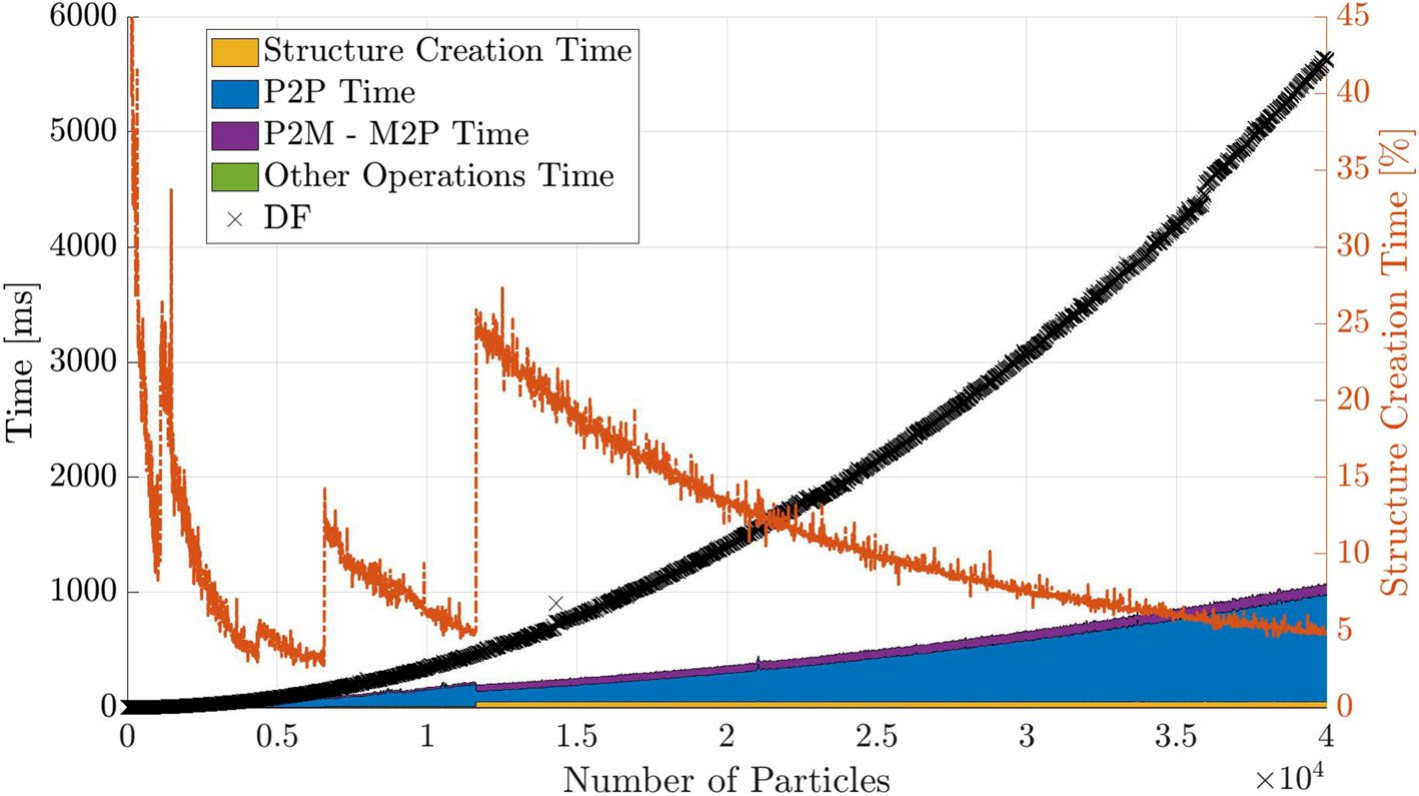
Computational time contributions for the FMM method with a number of multipole expansions of $p = 5$, separated into the different algorithmic steps. The right y-axis shows the contribution of the time needed to create the structure compared to the total time

The largest contribution is from the particle-to-particle (P2P) calculations which is essentially the bare-bones force calculation part of the algorithm. It is clearly visible that in the range of over $N = 10000$ particles, the FMM is significantly faster at calculating the particle interactions.

**Fig. 12:**
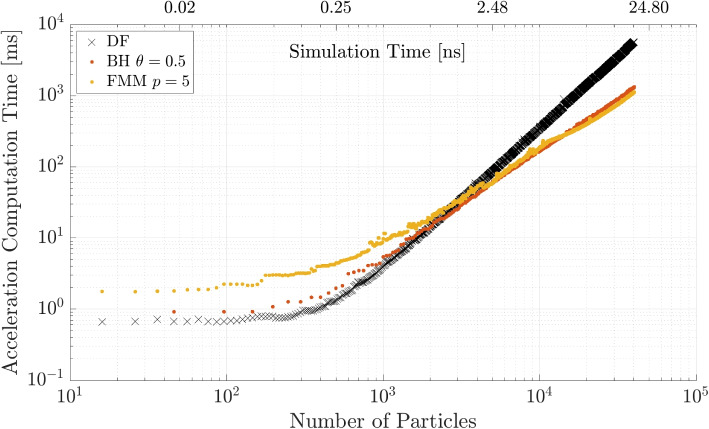
BH and FMM computation time for fixed parameters $\theta = 0.5$ and $p = 5$ with a comparison to the DF method

Lastly, the FMM and BH code are compared for the medium accuracy parameter of each parameter study to investigate at which point they outperform the other and the DF method. The results for the simulation are shown in Fig. 12. The BH method and FMM method intersect with the DF method line at the approximately same number of particles around $N = 3\times 10^{3}$. The FMM is faster than the BH code for an increasing number of particles.

The observed order of computations is compared to the theoretical order of the algorithms. The results from Fig. 12 are plotted with a linear scaling of the axis and a curve fit to the theoretical computational order is applied: $\mathcal {O}(N^2)$ for the DF method, $\mathcal {O}(NlogN)$ for the BH code, and $\mathcal {O}(N)$ for the FMM. The results are shown in the Appendix in Fig. 21 and Table 1. The fit of the data to the theoretical computational order is very accurate as showcased by the $R^2$ values and with the model and parameters *a* and $a_0$^4^ the computational time and total number of operations for higher numbers of particles can be accurately estimated.

**Table 1:** Curve fit results and goodness of fit parameter $R^2$ from MATLAB. Parameter *a* in [ms] and $a_0$ in [GFLOP]

Algorithm	Order $\mathcal {O}$	Fit type	Parameter *a*	Parameter $a_{0}$	$R^{2}$
DF	$N^2$	$a*N^2$	$3.37\times 10^{-6}$	$4.04\times 10^{-5}$	0.999
BH	*Nlog*(*N*)	$a*Nlog(N)$	0.007	0.084	0.9640
FMM	*N*	$a*N$	0.025	0.3	0.9724

### Accuracy/Error estimation

The simulations with the varying parameters from the previous section are used for the calculations of the $|L |^2$ norm.

A $|L |^2$ norm of $|L |^2 < 1\times 10^{-2}$ is a sensible lower bound of accuracy, since the leapfrog integrator with its second order accuracy is accurate to that level and further refinement in the accuracy of the force calculation would not improve the overall accuracy of the simulation.

The $|L |^2$ norm for accuracy in the acceleration is calculated from the error in the relative acceleration at a given simulation time $t_i$:

10 $$\begin{aligned} e_{rel}^{i} = \frac{\Vert \mathbf {a}^{i}_{DM}-\mathbf {a}^{i}_{OM}\Vert _{2}}{\Vert \mathbf {a}^{i}_{DM}\Vert _{2}} \end{aligned}$$

where OM (other method) stands for either the BH or FMM code. From this we can calculate the $|L |^2$ norm:

11 $$\begin{aligned} |L |^2= \sqrt{\frac{1}{{N}}\sum \limits _{i=1}^{N} e_{rel}^i} \end{aligned}$$

**Fig. 13:**
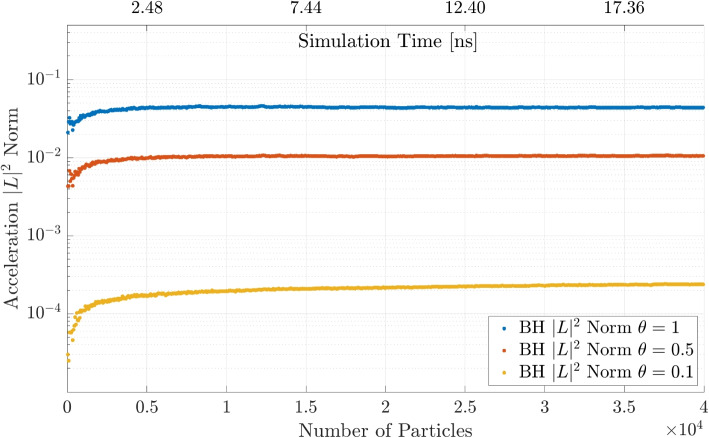
Acceleration $|L |^2$ norm comparison for Barnes-Hut method with different values of the MAC $\theta$

Figure 13 shows the norm for all particles in the simulation. This simulation compares the BH algorithm to the DF method for the same parameters $\theta$ from earlier. As expected the accuracy increases with lowering the tree acceptance angle; both $\theta = 0.1$ and $\theta = 0.5$ achieve an accuracy below the bound of $\epsilon < 1\times 10^{-2}$ and can therefore be considered as sufficiently accurate. Outliers with significantly higher error exist but do not bias the overall quality of the simulation.

**Fig. 14:**
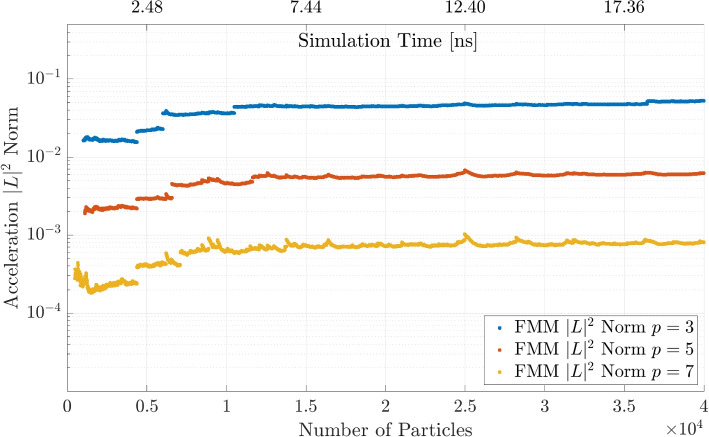
Acceleration $|L |^2$ norm comparison for Fast Multipole Method with different values of *p*

The results for the FMM are shown in Fig. 14. While the errors for the high $p = 7$ are higher than for the comparable BH simulation of $\theta = 0.1$, the $p = 5$ simulation shows very similar accuracy to the $\theta = 0.5$ simulation. The maximum outliers are also quite similar. For the FMM, both $p = 5$ and $p = 7$ achieve sufficiently accurate results while $p = 3$ exceeds the threshold of $\epsilon < 1\times 10^{-2}$ error in accuracy.

**Fig. 15:**
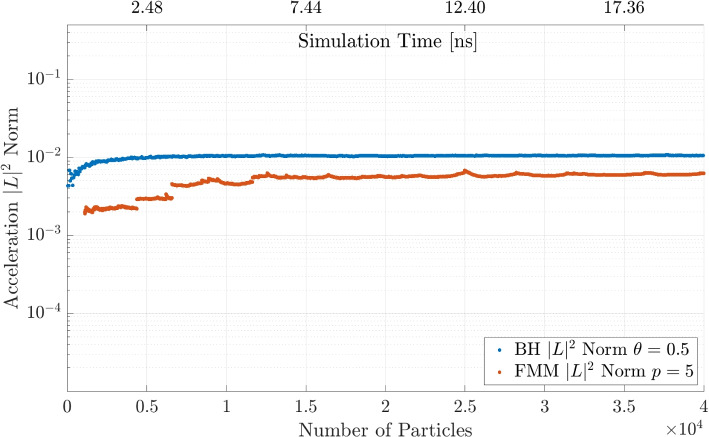
Acceleration $|L |^2$ norm comparison between the BH algorithm with $\theta = 0.5$ and the FMM with $p = 5$

In Fig. 15 the comparable BH and FMM simulations are shown. As expected, the FMM is slightly more accurate than the BH code for a similar amount of approximations defined by the parameters.

In theory, if $\theta \rightarrow 0$, the results of the BH tree code should converge to the ones of the DF method. Similarly when $p \rightarrow \infty$, the code should converge to the DF results. This is neither practical nor relevant, since the additional accuracy is irrelevant due to the integration error. The degree of approximation of far-field Coulombic force contributions is determined by the parameter of expansion, p.

Publications have proposed a simple formula to chose an appropriate parameter of expansion [14]:

12 $$\begin{aligned} p \ge -log_2(\epsilon ) \end{aligned}$$

where $\epsilon$ is the chosen accuracy. For $\epsilon = 10^{-2}$, the value for the multipole expansion parameter should be $p \ge 4.61$. This matches very well with the observation that the error drops below the required accuracy for $p = 5$, while $p = 3$ is not sufficient.

### Parameter variation

The dependence of the computational time and the $|L |^2$ on the tuning parameters is shown for the BH and FMM code. The computational time on the left y-axis is normalized by the computation time of the DF method. The horizontal line shows the intersection with the desired accuracy of $\epsilon =1\times 10^{-2}$.

**Fig. 16:**
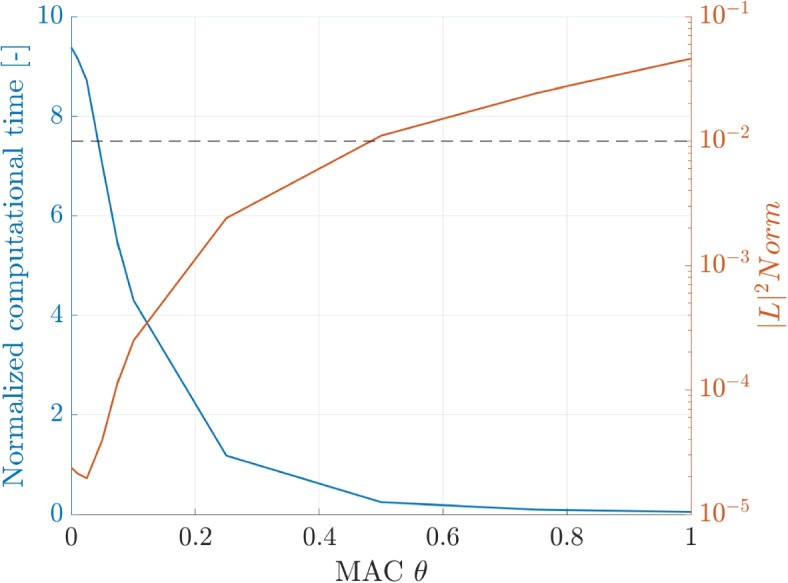
Dependence of the normalized computational time and $|L |^2$ norm on the MAC $\theta$

First, the MAC $\theta$ is shown in a range between $\theta = 0$ to $\theta = 1$ in Fig. 16. The trends show a decreasing computational time and an increasing error with increasing $\theta$ as previously shown. The computational time increases exponentially when lowering the MAC below 0.5, which was identified as the maximum $\theta$ to achieve the required accuracy.

**Fig. 17:**
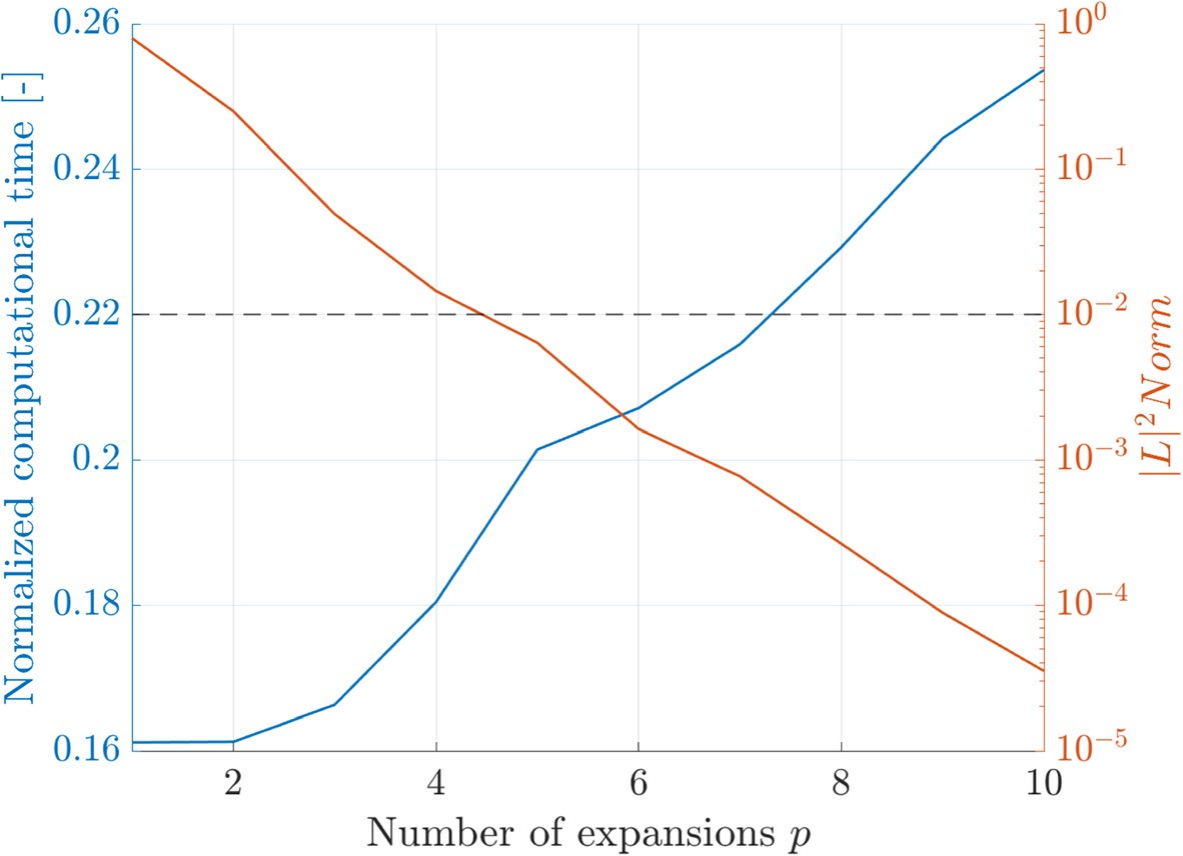
Dependence of the normalized computational time and $|L |^2$ norm on the number of expansions *p*

Second, the number of expansions *p* are varied in the range between $p = 1$ and $p = 10$ and the results are plotted in Fig. 17. The computational time increases and the error decreases with an increased *p*. Here, the dependence on the computational time is practically linear, while the $|L |^2$ norm follows a logarithmic trend.

The graphs can be used to chose the appropriate tuning parameter in the simulation for a prescribed accuracy. The number of expansions can be increased further but at a point the error will not decrease linearly anymore due to rounding errors and floating point computational accuracy.

### Energy conservation

**Fig. 18:**
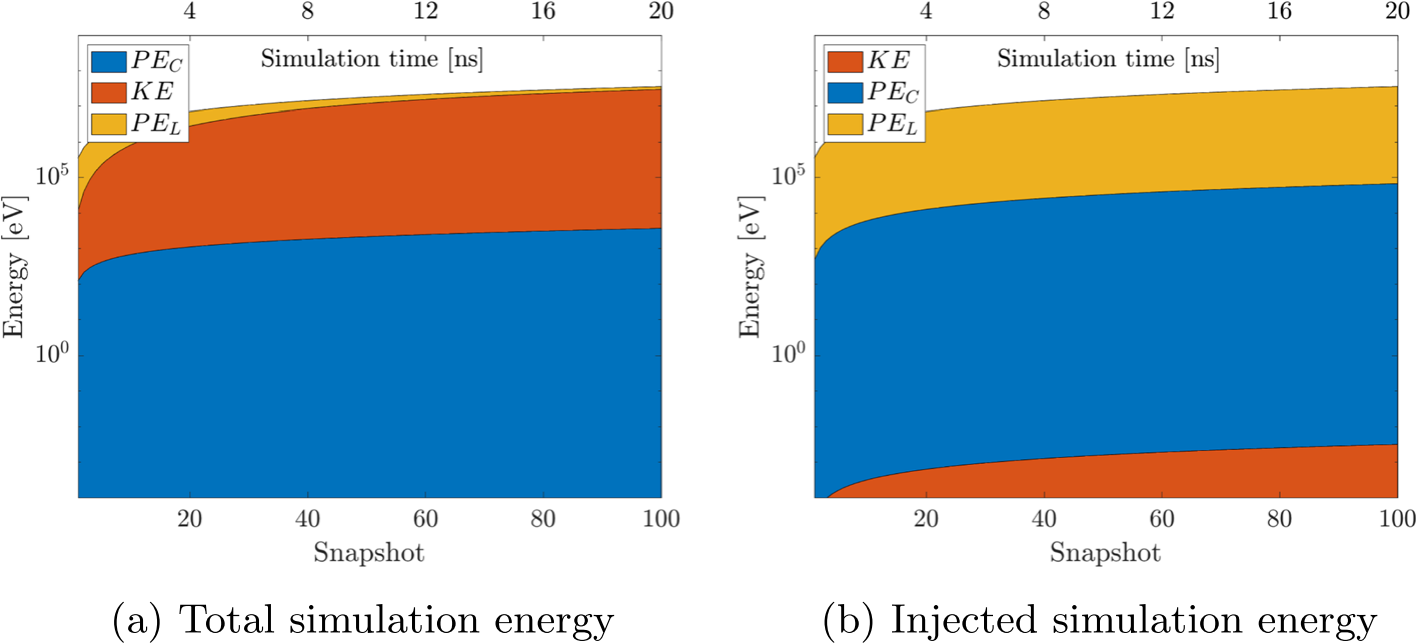
Different energy components for the DF simulations over the simulation time. The y-axis utilizes a logarithmic scaling so that all energy components are properly visible

Another important aspect to ensure the accuracy of the algorithms is energy conservation. Injecting new particles adds to the energy of the system. Therefore, the total energy of the simulation is compared to the injected energy to the simulation. This is repeated for all algorithms. The tuning parameters are $\theta = 0.5$ and $p = 5$, since they fulfill the accuracy requirement. The breakdown of the different energy components is shown in Fig. 18 for the DF simulation. $PE_C$ is the potential energy, *KE* the kinetic energy, and $PE_L$ the background electric field energy. Total simulation energy refers to the energy components of all the particles that are already within the simulation domain, while injected energy refers to the energies that is initially added when injecting the particles.

**Fig. 19:**
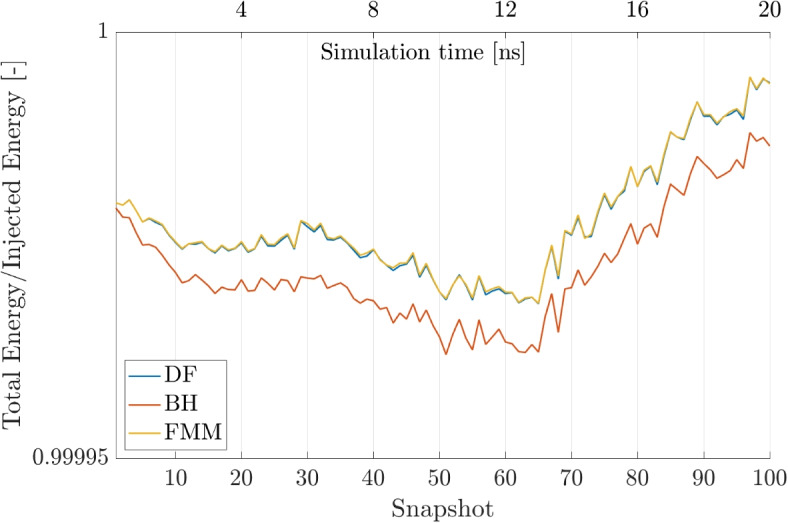
Ratio of Injected and Total Energy in the simulation for all three algorithms

Hence, energy is conserved if the ratio of the injected energy and the total energy in the simulation is approximately $\frac{E_{Injected}}{E_{Total}} \approx 1$. This ratio is plotted in Fig. 19 for the three algorithms and it can be clearly seen that the energy is conserved with less than a 0.1% error margin.

### Particle trajectories

**Fig. 20:**
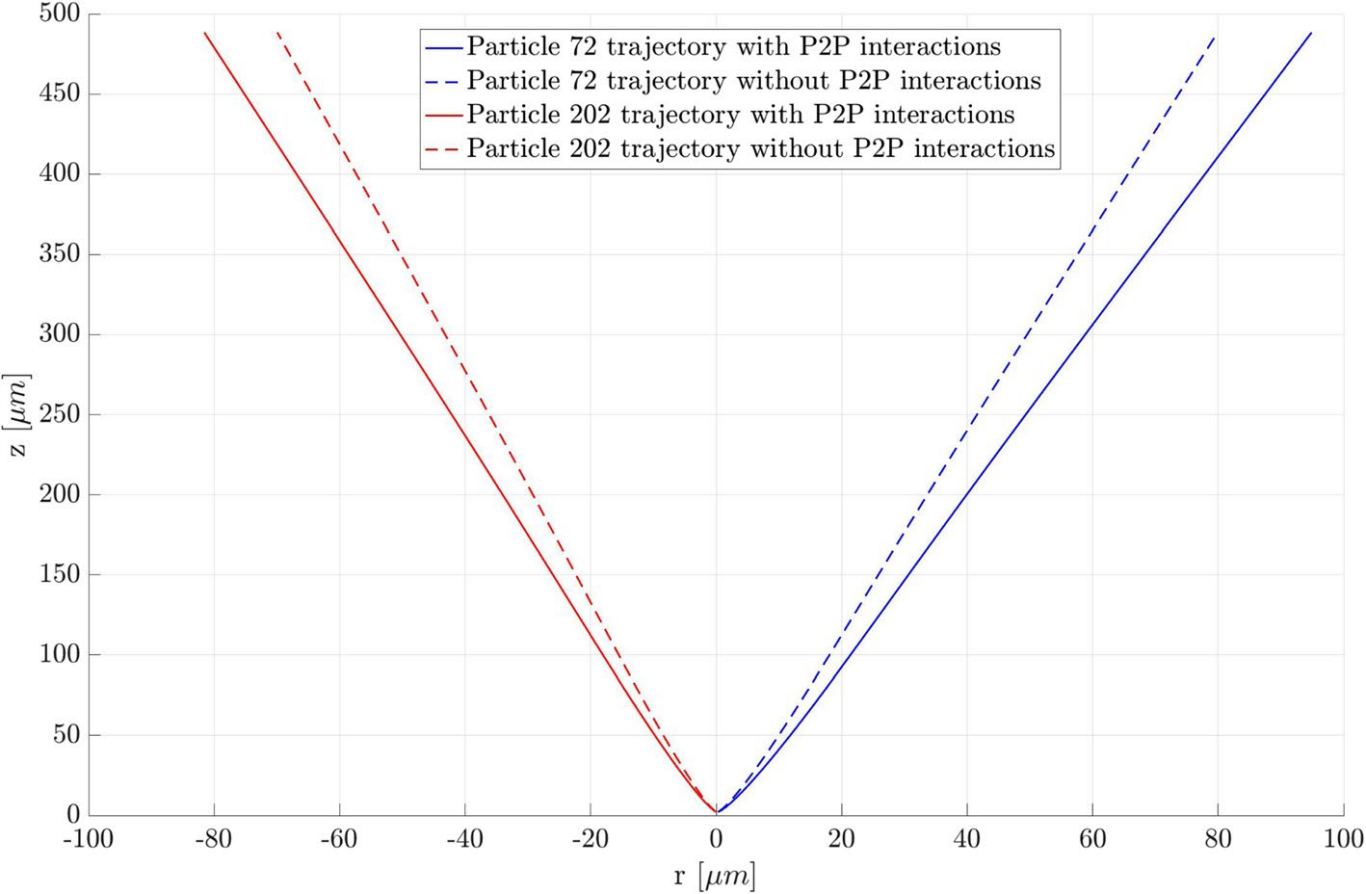
Particle trajectories in the rz-plane with and without P2P force calculations. For better visibility of the trajectories, one particle is shown to go to the left and one to the right. There is rotational symmetry around the z-axis

**Fig. 21:**
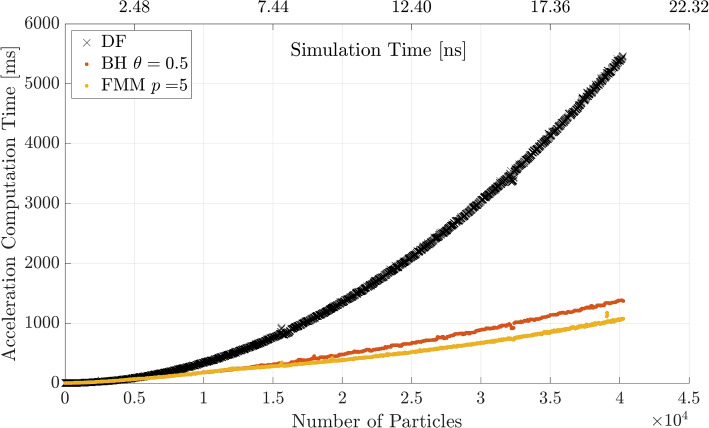
BH and FMM computation time for fixed parameters $\theta = 0.5$ and $p = 5$ with linear x-axis scaling and a comparison to the DF method

The use of faster algorithms is only necessary if the relative importance of the Poisson space charge effects is non-negligible compared to the Laplace background field. Especially in the vicinity of the emitter, where a high charged particle density is encountered, the effects cannot be neglected and particle-particle forces must be considered. An analysis was performed comparing the trajectories of two distinct particles when computed with only the background field, the DF algorithm, the BH algorithm, and the FMM. The initial conditions for those particles are all informed by the EHD model. The trajectories for two particles are shown in Fig. 20, once without a Poisson field calculation (dashed line) and with a Poisson field calculation. The particle trajectories were computed with all three algorithms but the differences in trajectories are below 0.1% and thus not visible in the figure. The particles were chosen because Particle 72 has the highest absolute initial XY-velocity, while Particle 202 has the lowest. For both particles the difference between the trajectories with and without the Poisson field is quite significant. There is a deviation (in the final radial position) of 17.4% in the final position of particle 72 and 15.5% in the final position of particle 202 respectively. These findings support the choice of an n-body simulation, as the inability to capture these particle-particle forces of the latter could produce similar errors in a PIC approach if the grid cannot be reduced to the order of the inter-particle spacing near the emission site.

The analysis indicates that even with a background electric field, the error in the particle trajectories without the computationally intensive particle-particle force calculations is high and thus the particle-particle forces need to be calculated. In general, if the simulation results are used as the initial conditions to a PIC simulation at a later stage, it is of utmost importance that these initial conditions are as precise as possible.

### Multi-threading effects and gpu computing

Multi-core computing has shown to achieve a tremendous increase in computational speed for different types of algorithms. For n-body simulations, GPUs which typically have a very high number of cores are used to accelerate computations for the three algorithms presented in this paper. Implementations of these algorithms on GPUs and data on their performance can be found in [24–26].

Oftentimes, for small numbers of particles in a simulation, the computational overhead for creating the threads is higher than the actual benefit to the computational time when using multiple cores. For simulations with high numbers of particles, where the approximation algorithms become faster than the direct method, the overhead is minimal compared to the force calculation time and multithreading can provide speedups of 100 time or more depending on the number of cores [26] in addition to the faster algorithm. This drastically expands the capabilities of the code when the memory allocation is adjusted as well. An example of a massive n-body simulation is the Department of Energy “Exascale” project where up to $10^{13}$ particles in a gravitational n-body code are simulated with GPU parallelizations [27].

One of the limitations of using an n-body approach on multiple processors is the transfer of data in distributed memory applications. While PIC can be highly optimized for distributed memory applications, it is much more complex for n-body codes. Research on how to optimize the fast algorithms for distributed memory applications is ongoing (an example implementation for the BH code can be found in [28], for the FMM in [29]). In conclusion, the n-body approach has been shown to be competitive to PIC in shared memory applications, which might not necessarily hold true for distributed memory applications.

## Conclusions and future work

Based on the simulations, the first important aspect is that the “fast” algorithms only become faster when simulating a minimum of $N = 1000 - 10000$ particles. After that, the computations are significantly faster (at $N=10,000$ by a factor of $f=1.35$ and at $N=100,000$ by a factor of $f=13.5$). In terms of the tuning parameters, if a second order Leapfrog integrator is used, a MAC of $\theta < 0.5$ or a number of multipole expansions of $p > 5$ is required to not introduce further numerical errors in addition to the integration error. The curve fits for the computational time (Table 1) can be used to extrapolate the computation time for physically relevant time scales ($> 1$ $\mathrm {\mu s}$).

A summary of the tuning parameter choice and total computation time comparison for a $\Delta t = 1$ $\mathrm {\mu s}$ simulation is shown in Table 2.

**Table 2:** Appropriate tuning parameter choice for a given accuracy $\epsilon$ and extrapolated simulation time ($\Delta t = 1$ $\mathrm {\mu s}$) for a $I=323$ nA simulation on the computer described in Section 4 with four parallel threads

**Metric**	**DF**	**BH**	**FMM**
Tuning Parameter ($\epsilon \le 0.01$)	-	$\theta \le 0.5$	$p \ge 5$
Computation Time	25,370 hrs	239 hrs	140 hrs

In this paper, two different algorithm updates have been presented to solve the electrospray plume propagation problem of an ionic liquid ion source. Up to one order of magnitude reduction of the computational time from the direct force calculation in the range of simulated particles $N \sim 10^5$. As shown previously, this is suitable for the number of particles that need to be simulated for an ionic liquid electrospray plume in steady state. The reduction in computational time is done at the expense of a minimal accuracy reduction, up to 0.1%. The trade-off between accuracy and time execution can be regulated with the parameters $\theta$ and *p* in the BH and FMM approaches respectively.

This study has shown that FMM has better execution time vs. accuracy relationship, and better scalability with the number of particles *N*. Conversely, the BH method offers slightly lower performance, but at the advantage of a much higher simplicity and ease of implementation.

A fast N-body approach is very appealing to solving particle propagation problems that were traditionally solved with Particle-In-Cell (PIC) methods. In a PIC approach, the effect of the electric field on other particles is computed by solving a Poisson equation in a grid. The accuracy of the PIC method is then regulated by the grid size, which can be very challenging to manage and require special treatment when dealing with close particle-particle interactions. This can be especially challenging with electrospray plume propagation problems, where the electric fields and particle dynamics span across multiple scales (slow $\sim 0.1$ eV near the emission region, $E \sim 10^9$ V/m, $\Delta \sim 3$ nm; fast $\sim 1500$ eV in the field free region, $E \sim 10^2$ V/m, $\Delta \sim 1$ mm) and $\Delta$ is the average distance between particles.

Even at larger scales (for instance, at the array level, or even spacecraft considerations), fast mesh free methods such as the ones presented in this paper can emerge as a very competitive methodology compared to PIC. At such scales, where the number of particles extends beyond $N \sim 10^6$ (about 100 arrays of particles emitting at 200 nA), the limitation of both the FMM and BH methods seems not longer to be computational scalability, but storage capability. In such cases, the user could still use this methodology with simplifications such as a macroparticle approach, and run at PIC’s level accuracy, at least. For this reason future research efforts will use these new algorithms to extend electrospray modeling to the array level.

## Figures and Tables

**Fig. 22:**
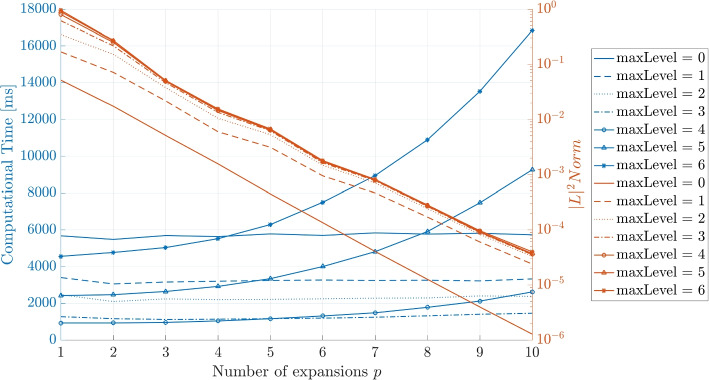
Dependence of the computational time and $|L |^2$ norm on the number of expansions *p* and the maxLevel parameter

## Data Availability

The data that support the findings of this study are available from the corresponding author upon reasonable request.

## Appendix A: additional figures

Figure 21 is mentioned within the paper. It uses a linear scaling on both axes and thus, the trends ($N^2$, *NlogN*, and *N*) in the computational time can be directly observed from the Figure.

Aside from the number of expansions, a second parameter in the FMM can be varied: maxLevel. Since its variation is outside the scope of this paper, the results are included here, in the Appendix, in Fig. 22.

The Figure shows the relation between the force computation time and $|L |^2$ norm against the number of expansions present in the FMM for different values of the maxLevel parameter. The maxLevel parameter is one of the tunable parameters in the code that affects the cell creation portion of the FMM, which can have a large impact on the computation time required.

For this implementation of the FMM, all particles in the simulation are covered with a set of base-level cubic cells, such that there are $n_x$ cells in the $\hat{x}$ dimension, $n_y$ cells in the $\hat{y}$ dimension, and $n_z$ cells in the $\hat{z}$ dimension, with at least one of $n_x$, $n_y$, or $n_z$ equal to 1. Given the symmetry of the electrospray simulation analyzed, typically $n_x = n_y = 1$. The maxLevel parameter then controls the number of times these base-level cubic cells are subdivided into octants, such that if $m = \text {maxLevel}$, the cubic cells will be subdivided into a set of $2^m \times 2^m \times 2^m$ subcells.

With fewer cells, the computation time is dominated by computing particle-particle interactions, hence the result that when maxLevel is equal to zero, the computation time is comparable to that of the direct force implementation. With more cells, the cell-cell interactions, such as the multipole to local, multipole to multipole, and local to local interactions, are performed more often and take up more of the computation time in the FMM algorithm. Additionally, the more cell-cell interactions performed, the larger the $|L |^2$ norm, due to the approximations in the multipole and local expansions propagating throughout the cell structure.

Figure 22 demonstrates the importance of the cell creation, the benefit of optimizing the maxLevel parameter, and how the optimal choice of the maxLevel parameter depends on the number of expansion terms used.

## Appendix B: detailed code implementation

An overview over the exact implementation of the Fast Multipole Method in the code is provided here:

**Pre-processing.** After the cell structures are created, the pre-processing takes place, which consits of sorting the particles into supercells and subcells, using a morton index, counting the number of filled cells, and allocating the proper amount of space for all multipole and local expansions and other variables required. Morton indices allow cells to be uniquely labeled in 3-D space using integer values, such that given a morton index, *m*, and the number of cells that make up the next highest level $n^3$, $\text {floor}(m / n^3)$ gives the index of the next highest level cell and $m\mod n^3$ gives the index of the cell relative to the next highest level cell [30].

**Particle to Particle.** The particle to particle (P2P) calculations are used at the lowest level of the cell structure. They compute the force between particles inside cells which are too close together for the multipole expansions to converge. For a cell, *A*, cells which are considered too close are those within a 3x3x3 box centered on *A*. See also [18] Section 8.1, number 6.

**Particle to Multipole.** The particle to multipole (P2M) calculations compute the multipole moments for the lowest level cells (Section 8.1, number 1 from [18]). Theorem 5.2 from [31] gives the equation for the multipole expansion coefficients, $M_n^m$, as well as convergence criterion.

**Multipole to Multipole.** After the multipole moments for the lowest level cell are calculated, they are passed upwards to larger cells(Section 8.1, number 2 from [18]). The multipole to multipole (M2M) step allows this propagation to occur and is analogous to the Barnes-Hut method, which computes the net charge and center of charge of a cell using the cells from the next lowest level.

**Multipole to Particle.** Since every cell has a multipole expansion associated with it, the treecode $\mathcal {O}(N\log {N})$ version can be completed using the multipole to particle (M2P) step (Section 5.2 from [31]). This is very similar to the Barnes-Hut method step in which the force is computed between particles and all cells not already accounted for. However, the multipole expansions have a well-defined convergence region, and are better approximations than the center of charge and net charge used in the Barnes-Hut method. Additionally, the multipole expansions require a little more work to convert into a force, because they give expansions of the potential fields, not the electric fields [32]

**Multipole to Local.** To continue with the FMM, the conversion from multipole expansions to local expansions (M2L) is critical to achieve the $\mathcal {O}(N)$ behavior. In this step, those cells which are within a 3x3x3 box centered on the parent cell, but outside of a 3x3x3 box centered on the cell being considered, have their multipole moments converted into a local expansion within the cell being considered (Section 8.1, number 3 from [18]). The local expansions within each cell give the contribution to electric potential due to the multipole moments from the cells that are interacted with in this step.

Note that in this step, the code uses the $\mathcal {O}(p^3)$ method of rotating coordinate systems using Wigner rotation matrices in order to improve the computation time over the baseline $\mathcal {O}(p^4)$ solution, where *p* is the number of expansion terms retained in the multipole/local expansions. This method is discussed near the end of Section 5.3 in [31], also in Section 5 of [18], and implemented in the code from https://github.com/barbagroup/gemsfmm.

**Local to Local.** After each cell has a local expansion associated with it, the local expansions are passed to smaller cells in the local to local (L2L) step. While the multipole expansions are propagated to larger cells, the local expansions are propagated to smaller cells. The goal for this step is to arrive at local expansions for the smallest cells which will contain information about the electric potential due to all cells not included in the P2P calculations (Section 8.1, number 4 [18]).

**Local to Particle.** The final step is to evaluate the local expansions of the potential in each cell at the positions of each of the particles inside it to compute the force. This is accomplished in the local to particle (L2P) step (Section 8.1, number 5 [18]). Also, note that there are some conversions involved to convert the potential into the electric field, as in the M2P step (Appendix D [18]).

**Additional Notes.** To prevent rounding errors, the multipole and local expansions are scaled, as in Section 4 of [18].

## Footnotes

1 The $\mathcal {O}$-notation refers to the computational time complexity, not spatial complexity, every time it is used in this work. Further information about the use of the Big $\mathcal {O}$ notation in computer science can be found in [33].

2 https://github.com/barbagroup/gemsfmm

3 The jump discontinuities that are observed in the components of the computation time in Fig. 11 are due to another level being added to the octree structure. If *n* is the number of levels, then we have $8^n$ nodes in the octree. This behavior results in oscillations of the computation time around a linear slope which can be better observed in Fig. 5 in the appendix of reference [11]. The location of the jump discontinuities depends on parameter *p* and its effects can also be seen in Fig. 14, since the amount of approximations made increases as a consequence of the modified tree structure. Overall, the scaling of the computation time with $\mathcal {O}(N)$ is not affected by the changes in the tree structure though.

4 Parameter *a* depends on the hardware of the computer and is only valid for our specific computing station. Parameter $a_0$ is given by $a*c_{CPU}$ where $c_{CPU}$ is a hardware specific parameter, which represents the number of double-precision floating point operations per second that the CPU performs. The value should be available in the data sheet of the processor. For the i7-6500U the parameter $c_{CPU} \approx 12$ GFLOP/s [34]. With $a_0$ and $c_{CPU}$, an estimated computation time can be obtained for different CPUs. It should be noted that this is just a rough estimate.

## Code availability

The SOLVEiT (Simulating the Operational Local Volume of Electrospray ion Thrusters) code was made available for open source use under the following GitHub repository and is subject to updates: https://github.com/AstraLab-Cornell/SOLVEiT-Code.

## References

[1] Krejci D, Mier-Hicks F, Fucetola C, Lozano P, Schouten AH, Martel F (2015) Design and Characterization of a Scalable ion Electrospray Propulsion System. Joint Conference of 30th ISTS, 34th IEPC and 6th NSAT, Hyogo-Kobe, Japan pp 1–11. https://www.researchgate.net/publication/280098850. Accessed 15 June 2022

[2] Gamero-Castaño M, Hruby V (2001). Electrospray as a source of nanoparticles for efficient colloid thrusters. J Propuls Power.

[3] Iribarne JV, Thomson BA (1976). On the evaporation of small ions from charged droplets. J Chem Phys.

[4] Thuppul A, Wright P, Wirz R (2018). Lifetime considerations and estimation for electrospray thrusters..

[5] Cidoncha XG, Lozano PC, Bendimerad R, Petro EM, Hampl SK (2022) Modeling and Characterization of Electrospray Propellant-Surface Interactions, 2022 IEEE Aerospace Conference (AERO). p 1–11. 10.1109/AERO53065.2022.9843583

[6] Gallud X, Lozano PC (2022). The emission properties, structure and stability of ionic liquid menisci undergoing electrically assisted ion evaporation. J Fluid Mech.

[7] Petro EM, Gallud X, Hampl SK, Schroeder M, Geiger C, Lozano PC (2022). Multiscale modeling of electrospray ion emission. J Appl Phys.

[8] Nuwal N, Azevedo VA, Klosterman MR, Budaraju S, Levin DA, Rovey JL (2021). Multiscale modeling of fragmentation in an electrospray plume. J Appl Phys.

[9] Asher J, Huang Z, Cui C, Wang J (2022). Multi-scale modeling of ionic electrospray emission. J Appl Phys.

[10] Barnes J, Hut P (1986). A hierarchical O(N log N) force-calculation algorithm. Nature.

[11] Yokota R, Barba L (2010) Treecode and fast multipole method for n-body simulation with cuda

[12] Fortin P, Athanassoula E, Lambert JC (2011) Comparisons of different codes for galactic n-body simulations. Astron Astrophys 531. 10.1051/0004-6361/201015933

[13] Yokota R, Barba LA (2012) Parameter tuning of a hybrid treecode-fmm on gpus

[14] Dehnen W (2014) A fast multipole method for stellar dynamics. Comput Astrophys Cosmol 1(1). 10.1186/s40668-014-0001-7

[15] Krejci D, Lozano P (2017) Micro-machined ionic liquid electrospray thrusters for cubesat applications

[16] Coffman CS, Martínez-Sánchez M, Lozano PC (2019). Electrohydrodynamics of an ionic liquid meniscus during evaporation of ions in a regime of high electric field. Phys Rev E.

[17] Press WH, Teukolsky SA, Vetterling WT, Flannery BP (1992). Numerical Recipes in C.

[18] Kurzak JPB (2006). Fast multipole methods for particle dynamics. Mol Simul.

[19] Waltz J, Page G, Milder S, Wallin J, Antunes A (2002). A performance comparison of tree data structures for n-body simulation. J Comput Phys.

[20] Aarseth S (2009). Gravitational N-Body Simulations: Tools and Algorithms.

[21] White CA, Head-Gordon M (1994). Derivation and efficient implementation of the fast multipole method. J Chem Phys.

[22] Board J, Schulten L (2000). The fast multipole algorithm. Comput Sci Eng.

[23] Cheng H, Greengard L, Rokhlin V (1999) A fast adaptive multipole algorithm in three dimensions. J Computat Phys 155(2):468–498. 10.1006/jcph.1999.6355

[24] Darve E, Cecka C, Takahashi T (2011). The fast multipole method on parallel clusters, multicore processors, and graphics processing units. C R Mécanique.

[25] Lashuk I, Chandramowlishwaran A, Langston H, Nguyen TA, Sampath R, Shringarpure A, Vuduc R, Ying L, Zorin D, Biros G (2009) A massively parallel adaptive fast-multipole method on heterogeneous architectures. In: Proceedings of the Conference on High Performance Computing Networking, Storage and Analysis, pp 1–12. 10.1145/1654059.1654118

[26] Belleman RG, Bédorf J, Zwart SFP (2008). High performance direct gravitational n-body simulations on graphics processing units II: An implementation in CUDA. New Astron.

[27] Alexander F, Almgren A, Bell J, Bhattacharjee A, Chen J, Colella P, Daniel D, DeSlippe J, Diachin L, Draeger E, Dubey A, Dunning T, Evans T, Foster I, Francois M, Germann T, Gordon M, Habib S, Halappanavar M, Hamilton S, Hart W, (Henry) Huang Z, Hungerford A, Kasen D, Kent PRC, Kolev T, Kothe DB, Kronfeld A, Luo Y, Mackenzie P, McCallen D, Messer B, Mniszewski S, Oehmen C, Perazzo A, Perez D, Richards D, Rider WJ, Rieben R, Roche K, Siegel A, Sprague M, Steefel C, Stevens R, Syamlal M, Taylor M, Turner J, Vay JL, Voter AF, Windus TL, Yelick K, (2020) Exascale applications: skin in the game. Philosophical Transactions of the Royal Society A: Mathematical, Physical and Engineering Sciences 378(2166):20190056. 10.1098/rsta.2019.0056 10.1098/rsta.2019.0056 PMC7015298 31955678

[28] Makino J (2004). A Fast Parallel Treecode with GRAPE. Publ Astron Soc Jpn.

[29] Kurzak J, Pettitt BM (2005) Massively parallel implementation of a fast multipole method for distributed memory machines. J Parallel Distrib Comput 65(7). 10.1016/j.jpdc.2005.02.001

[30] Bern M, Eppstein D, Teng Sh, Goodrich C (2001) Parallel construction of quadtrees and quality triangulations. vol 9. 10.1007/3-540-57155-8_247

[31] Beatson R, Greengard L (1997). A short course on fast multipole methods Numerical Mathematics and Scientific Computation.

[32] Rankin WT, Board JA (1999) Efficient parallel implementations of multipole based n-body algorithms. PhD thesis, USA, aAI9928860

[33] Cormen TH, Leiserson CE, Rivest RL, Stein C (2009). Introduction to Algorithms, Third Edition.

[34] Berkley SETI@Home (2022) Cpu performance. https://setiathome.berkeley.edu/cpu_list.php. Accessed 19 Aug 2022

